# Media framing of agricultural technology adoption in developing countries: Evidence from Juncao technology news coverage

**DOI:** 10.1371/journal.pone.0343652

**Published:** 2026-09-01

**Authors:** Ling Huang, Yunqing Zhang

**Affiliations:** International College, Fujian Agriculture and Forestry University, Fuzhou, China; Southern Federal University Academy of Biology and Biotechnology named after D I Ivanovsky: Uznyj federal’nyj universitet Akademia biologii i biotehnologii im D I Ivanovskogo, RUSSIAN FEDERATION

## Abstract

Juncao technology, a key agricultural technology innovation for Sustainable Development Goals, is praised for boosting agricultural yields, reducing poverty, and restoring ecosystems, yet how media framing shapes adoption remains unclear. This study employs BERTopic for theme mining and NRC Emotion Lexicon for emotion analysis to examine English-language news coverage of Juncao technology in 13 developing countries, drawing on a self-built corpus published between 2019 and 2025. The BERTopic analysis identifies five dominant topics: government-led rice cultivation and farmer training for sustainable production; local agricultural development, ecological management, and poverty alleviation; the Belt and Road Initiative—international agricultural investment for a shared global community; provincial and intergovernmental official visits; and contributions to the UN SDGs through livestock production. Emotion analysis shows that positive emotions prevail, with Trust and Anticipation most salient. Trust is closely associated with international cooperation, particularly within the Belt and Road Initiative, while Anticipation reflects expectations of improved agricultural production and economic gains. Negative emotions target existing challenges such as poverty, food insecurity, and environmental degradation rather than Juncao itself, positioning the technology as a solution. These findings demonstrate that emotional framing plays a key role in shaping perceived advantages and fostering adoption of technology.

## 1. Introduction

Agricultural technologies have always been recognized by governments and development organizations as effective instruments for improving farm productivity and alleviating poverty in developing countries. The continuous development and widespread dissemination of novel agricultural technologies are thus considered fundamental for achieving sustainable development [1]. It is broadly acknowledged that the responsible advancement of agriculture is essential to addressing, at a minimum, Sustainable Development Goals (SDGs) 1 (no poverty), 2 (zero hunger), 3 (good health and well-being), and significantly contributes to Goals 10 (reduced inequalities), 12 (responsible consumption and production), 13 (climate action), 14 (life below water), and 15 (life on land) [2]. However, the successful adoption of appropriate agricultural innovations by smallholder farmers remains a critical challenge, necessitating a deeper understanding of the factors affecting adoption, the perceptions of adopters and the effectiveness of technology dissemination.

Among various agricultural innovations, Juncao technology, developed by Professor Lin Zhanxi and promoted by the Chinese government, is a priority project that the United Nations Department of Economic and Social Affairs (UN DESA) actively supports. Juncao technology refers to techniques using herbaceous plants (Juncao grass) as a substrate for cultivating edible and medicinal fungi, thereby eliminating the need to cut down trees and preserving forest ecosystems [3]. Beyond mushroom cultivation, Juncao grass serves as a nutritious animal feed, significantly increasing livestock productivity, and is also adopted for soil and water conservation, fighting against desertification, preventing land degradation, and enhancing biodiversity [4]. Through technical assistance, expert deployment, and international cooperation, this technology has made significant contributions to food security, poverty alleviation, and environmental protection in developing countries [5]. Juncao technology, an integral part of China’s foreign aid, not only showcases China’s achievements in agricultural innovation but also carries the mission of promoting economic development and improving livelihoods in recipient countries. In the era of globalization, the global dissemination of Juncao technology functions as a bridge for technological exchange and cultural communication, which gets the world to recognize China’s technological value and development ideas.

According to the report by the Ministry of Foreign Affairs (MFA) of the People’s Republic of China [6], as a significant agricultural innovation, Juncao technology has been promoted in over 100 countries since its inclusion as a UN Development Programme priority cooperation project in 1994. More than 10,000 people have been trained and more than a dozen international training and demonstration bases have been established across Asia, Africa, and the Pacific, earning the technology recognition as a model of South-South cooperation. Past studies have almost exclusively focused on its technical development and diverse economic and ecological value, such as its use as a substrate for fungi, a cost-effective livestock feed, a tool for soil conservation or serving as biomass energy [7–10]. However, few studies explore the emotional adoption of Juncao technology concerning its realistic performance [11], particularly from a media perception perspective. Therefore, this study aims to complement prior research through providing a different perspective on the adoption of Juncao technology based on the news media data to uncover the latent insights. A corpus of news coverage was built from the developing countries on Juncao technology, employing both quantitative and qualitative analysis to examine its adoption. The purpose is to discuss measurable and descriptive aspects of emotions during Juncao technology’s diffusion. And the findings will offer invaluable insights for policymakers and international development agencies. By revealing the dominant narratives, key themes, and emotional undercurrents in media coverage, this study will provide recommendations to design more effective communication strategies, address potential public concerns, and ultimately expedite the global diffusion of agricultural innovations, as exemplified by Juncao technology, in regions where it is most needed.

## 2. Literature review

### 2.1 Juncao technology and sustainable development

**Fig 1:**
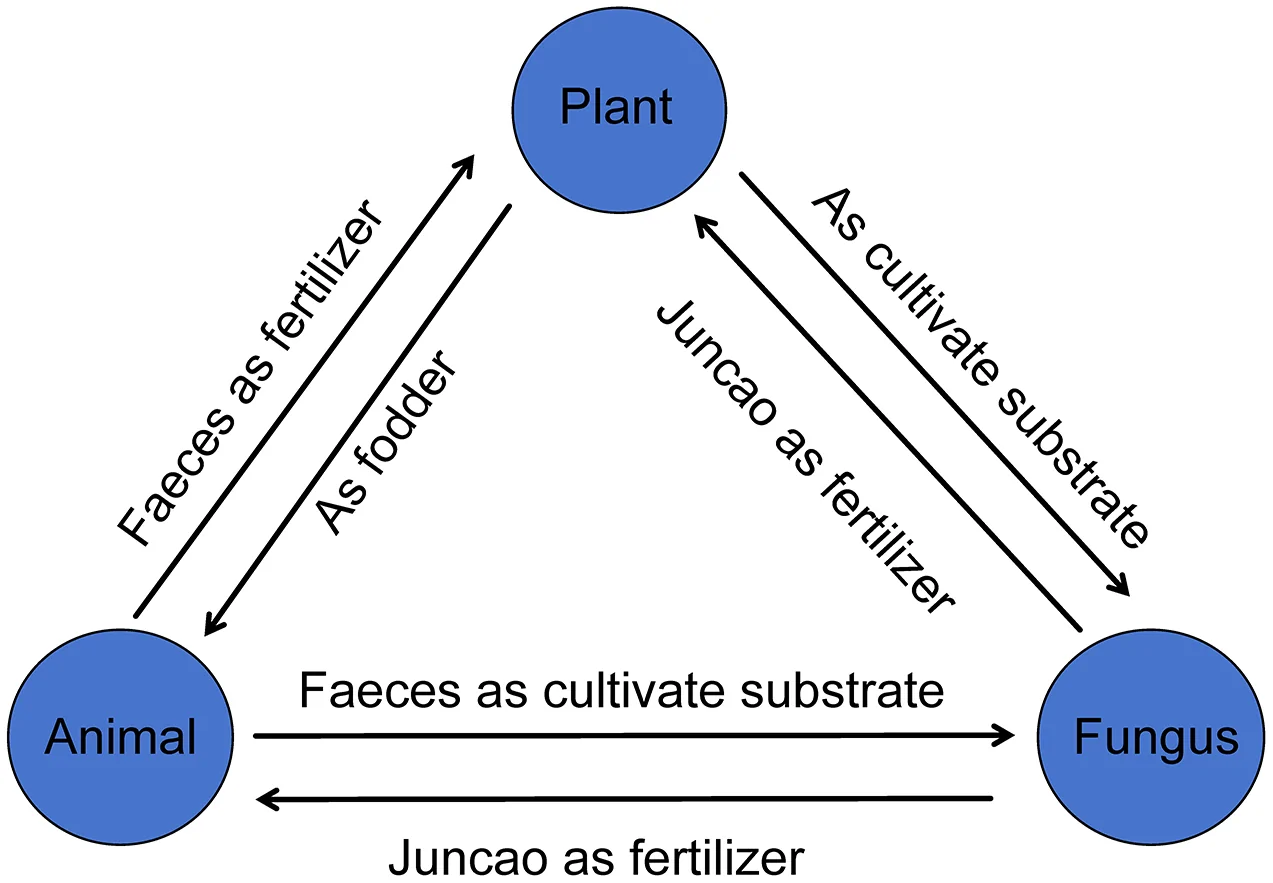
The nutrient recycling system of Juncao industry.

China is the country with the longest history of the use and artificial cultivation of edible and medicinal fungi. At present, there are more than 200 species of edible and medicinal fungi, such as *Lentinus edodes, Auricularia auricula and ganoderma lucidum*, widely cultivated worldwide, most of which originated in China [12]. The traditional cultivation of edible and medicinal fungi uses wood sawdust from broad-leaved trees as raw materials, thereby creating a conflict between the development of fungus production and forest ecological conservation. To resolve this global issue, Juncao technology was developed for cultivating edible and medicinal fungi using Juncao grass; research on the technology began in 1983 and achieved initial success by 1986 [4]. The word Juncao is derived from the Chinese 菌草 with *jun* meaning “fungus” and *cao* meaning “grass”, and has been applied to describe a range of forage species [13]. Over the past three decades, with strong support from the Chinese government, the scientific applications of Juncao technology have become increasingly diversified. Beyond fungi cultivation, its uses have been expanded to include renewable biomass fuel and environmental management in ecologically vulnerable regions [7]. Recent publications have also reported its superior adaptability and production potential such as drought tolerance [14] and greater biomass production [13]. Therefore, the application of Juncao technology promotes the development of the Juncao industry by creating an integrated livestock–fungi–plant system (see Fig 1) [8].

At present, Juncao technology has been disseminated in many developing countries (e.g., Papua New Guinea, Madagascar, Thailand, Laos, Fiji, Rwanda, Lesotho) as part of China’s foreign aid and UN cooperation initiatives. The global dissemination of Juncao technology is explicitly linked to the UN’s 2030 Agenda for Sustainable Development, providing a comprehensive solution that positively contributes to at least 13 SDGs [15]. By creating new income-generating opportunities for smallholder farmers and rural communities, it directly addresses SDG 1 (No Poverty) and SDG 2 (Zero Hunger) [16], while its role in reducing deforestation and rehabilitating degraded land aligns with SDG 15 (Life on Land). Recognizing its potential, major international bodies such as the UN DESA, the Food and Agriculture Organization (FAO), and the World Food Programme (WFP) actively support its application through South-South and triangular cooperation to help developing countries enhance their independent development capabilities. This establishes Juncao technology not merely as a technical innovation but as a comprehensive socio-economic intervention, with notable successes in improving livelihoods and ecological conditions in countries such as Fiji, Papua New Guinea, and Rwanda [5,6]. This model of cooperation is characterized by (1) achieving supply-demand matching through Juncao Technology cooperation projects; (2) sharing Juncao’s poverty alleviation experience and conducting various exchanges globally; (3) realizing the diverse, development-oriented benefits of the Juncao industry; and (4) emphasizing localized practices by tailoring Juncao foreign aid to specific local conditions [16]. Considering its significant implications for global development, the public and media perception of its adoption have become a critical area of inquiry and require further exploration.

### 2.2 Agricultural technology adoption in developing countries

Research on agricultural technology adoption has evolved significantly, particularly in understanding the complex interplay of factors influencing farmers’ decisions in developing countries [17]. Earlier studies focused on the adoption of natural resource management practices [18], improved varieties [19], chemical inputs [20], mechanization and infrastructure [21]. Meanwhile, recent meta-analyses indicate that there are three paradigms for exploring agricultural technology adoption: the economic constraints paradigm, the adopter perception paradigm and the innovation-diffusion paradigm [2]. The first paradigm highlights how economic factors, such as perceived profitability, access to credit, and market linkages, remain crucial to the adoption of modern agricultural technologies [22]. The second paradigm indicates farmers’ perceived needs and the perceived attributes of innovations that determine adoption behaviour [23]. It explores intrinsic factors such as farms’ age, income, knowledge, perceptions, and attitudes [24,25], as well as extrinsic factors of environment and context [26].

Meanwhile, the third paradigm is based on the pioneering work of Ryan and Gross [27], and was further developed by Feder et al. [28] and Rogers [29]. Rogers defined adoption as a decision of “full use of an innovation as the best course of action available” [29] (p.177), while emphasizing that the adoption of an innovation is a social process influenced by the perceived attributes of the innovation itself, communication channels, time, and the social system [30]. Communication channels are particularly critical and are divided into two main types [31]. Mass media channels, such as news media, are essential during the initial “knowledge stage” for making a broad audience aware of a technology’s existence [29]. The media enable the rapid dissemination of knowledge and information to a large audience and are capable of changing audience attitudes [32]. Following this, interpersonal communication becomes more powerful in the “persuasion stage” by influencing attitudes. Furthermore, Rogers [29] classifies individuals within a social system into five categories based on their innovativeness: innovators, early adopters, early majority, late majority, and laggards. Finally, the rate of adoption is strongly predicted by an innovation’s five perceived attributes. Specifically, relative advantage, compatibility, trialability, and observability are all positively correlated with faster adoption, whereas complexity is negatively correlated. Nevertheless, while these cognitive-functional attributes are instrumental in predicting adoption patterns, recent research emphasizes that such focus often overlooks social expressions of emotions in shaping the innovation-decision process [11]. The influence of various factors on adoption can therefore be understood within the three paradigms discussed above [2,31].

Furthermore, a recent trend reflects the growing focus on interpersonal communication and extension of agricultural technology from the perspective of public perception [33]. Consequently, a new approach has emerged that analyzes news media and social media discourse to explore public attitudes. For instance, studies increasingly employ sentiment and emotion analysis to examine innovations such as smart agriculture [34], revealing how media narratives and emotional tones can significantly influence adoption [35]. This underscores the media’s crucial role in shaping public discourse surrounding new agricultural technologies, such as Juncao technology, thereby impacting their adoption pathways [32].

### 2.3 Emotion analysis and its application

#### 2.3.1 Emotion and emotion analysis.

Emotion is a multidimensional construct that is complex and dynamic. From a psychological perspective, emotion is viewed as a psycho-physiological process comprising subjective feelings, cognitive appraisal, and bodily responses to internal or external stimuli [36,37]. In linguistics, investigations of emotion are based on diverse theoretical frameworks [38], including appraisal [39] and stance [40]. These theoretical frameworks usually employ textual analysis and empirical case studies to examine attitudes, stances, and judgments in specific contexts.

In recent years, computational linguistics (CL) has gained considerable attention, with one of its key focuses being the extraction of lexical, syntactic, and contextual emotion-related information from large-scale textual data [41]. CL is often linked to natural language processing (NLP), which utilizes computational techniques to extract human emotions from textual documents, facilitating the extraction and retrieval of information from such data [42]. Emotion analysis now moves beyond simple polarity classifications (positive, neutral, negative) to identify specific affective states, such as anger, happiness, and sadness. These specific emotions provide various insights into underlying psychological states. Generally, emotion analysis can be classified into two categories: dimensional models and categorical models [43]. Dimensional models, such as Russell’s Circumplex Model and the Valence-Arousal-Dominance (VAD/PAD) framework, quantify emotions along continuous axes, allowing for the assessment of intensity and similarity [44,45]. Categorical models, by contrast, propose discrete fundamental emotions, including Ekman’s [46] “Big Six” (Happiness, Sadness, Fear, Disgust, Anger, and Surprise) and Plutchik’s [47] “Wheel of Emotions” (Joy, Sadness, Anger, Fear, Disgust, Trust, Surprise, and Anticipation), illustrating their hierarchical relationships and intensities [48]. These models seek to represent, in computer systems, how beliefs and perceptions affect emotional reactions to events.

The NRC Emotion Lexicon (EmoLex) is a widely adopted resource for categorical emotion analysis. Developed at the National Research Council of Canada by Mohammad and Turney [48,49], EmoLex was constructed through large-scale crowdsourcing to associate more than 14,000 English lemmas with eight emotions grounded in Plutchik’s model, as well as positive/negative sentiment labels. The lexicon has since been expanded and adapted, including intensity-oriented variants and multilingual versions that enable applications across diverse languages and media ecosystems. Its core appeal lies in interpretability and portability: researchers can compute document-level or corpus-level emotion profiles by counting and normalizing emotion-bearing tokens, compare emotion prevalence across topics, outlets, or time, and communicate results in a manner that is transparent to non-technical audiences [50].

#### 2.3.2 The application of emotion analysis.

In recent years, emotion analysis tools and resources, such as EmoLex, have been applied to analyze user reviews, and support tasks such as crisis monitoring, policy communication, product feedback, and cross-cultural comparisons [51,52]. In addition, it has been adopted to analyse the public and individual attitudes and emotions in fields such as film review, news coverage, and technology diffusion from the perception perspective [53,54]. For example, Na et al. [55] focused on the online user comments on energy-saving products to explore emotional states, while Jiang [41] mined themes, emotions, and stance in the news coverage of the Russia–Ukraine War. In agriculture, studies using social media platforms such as Twitter, have examined direct user interactions and found predominantly positive emotions towards agricultural technology, influenced by engagement and positive language [56]. Ofori and El-Gayar [34] also adopted both emotion and content analysis to explore the drivers and challenges of precision agriculture adoption.

Although emotion analysis has gained growing attention in media studies [35], little is known about news coverage of specific agricultural technologies using such methods, particularly regarding the adoption of Juncao technology in developing countries. This gap limits a comprehensive understanding of the emotional mechanism, through which media may promote or hinder the adoption of specific agricultural technologies in these regions. Therefore, this study aims to fill this gap by conducting an emotion analysis of news media coverage of Juncao technology adoption in developing countries, using advanced text mining and emotion analysis techniques. The following two questions are to be addressed:

(1) What topics do the news media in developing countries focus on when reporting Juncao technology?
(2) What emotional features characterize news media coverage of Juncao technology in developing countries?

## 3. Method

**Fig 2:**
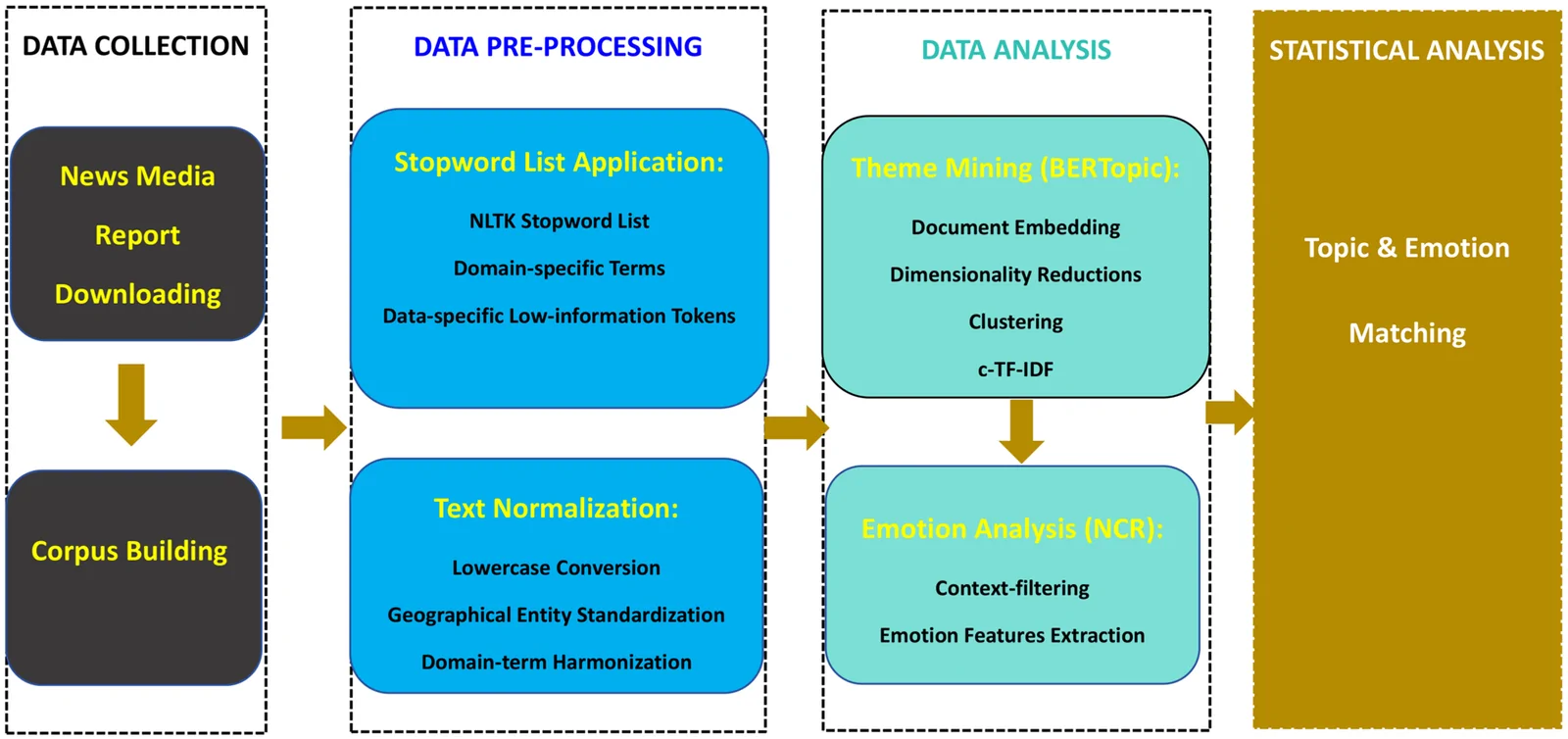
The methodological framework.

The research process, including text pre-processing, topic modeling, and emotion analysis, is summarized in Fig 2. The process initiates with data collection, where a specialized corpus of Global South news media was constructed to provide the empirical basis. This is followed by a rigorous data pre-processing stage to ensure data quality and reduce lexical noise. Subsequently, the stage of data analysis is conducted through two complementary strands: topic modeling (BERTopic) to identify latent thematic structures, and emotion analysis to quantify the affective framing of the discourse. Finally, statistical analysis is employed to validate the variations in emotional features across different topics, ensuring a robust triangulation between thematic content and affective framing.

### 3.1 Data collection

To investigate the dissemination and acceptance of Juncao technology, we constructed a self-built corpus comprising English-language news reports from media outlets in Global South countries that are primary recipients of the technology. Using “Juncao technology” as the key search term, we collected 97 news reports published between 2019 and 2025, totaling 55,216 tokens. The reports originate from 13 countries across four continents — Africa (Ethiopia, Tanzania, South Africa, Rwanda, Botswana, Kenya), Asia (Malaysia, the Philippines), Oceania (Papua New Guinea, Fiji, Vanuatu), and the Americas (Jamaica, Guyana). Collectively, these countries account for an estimated 514 million people, representing roughly 6–7% of the global population, and range from small island states to large African nations. This wide geographic and demographic coverage provides a representative view of media discourse on agricultural technology adoption in developing regions. The detailed composition of the corpus is presented in Table 1.

**Table 1:** Description of the corpus.

Country	Media	Number of Reports	Token
Papua New Guinea	The National	7	3301
	Post Courier	40	16926
Fiji	Fiji Government	3	1594
Malaysia	The Star	7	4075
Philippines	Philippine News Agency	1	425
Rwanda	The New Times	11	9886
South Africa	Independent Online	6	3020
Jamaica	Our Today	14	9239
Vanuatu	Vanuatu Daily Post	1	479
Guyana	Guyana Chronicle	1	1924
Ethiopia	Ethiopian Monitor	1	232
	The Ethiopian Herald	1	387
	Ethiopian News Agency	1	254
Tanzania	Tanzania Daily News	1	1372
Botswana	Daily News	1	1854
Kenya	Kenya News Agency	1	538
Total	13	97	55216

### 3.2 BERTopic

To identify the iterating key concepts and in turn hidden themes in our corpus, the Natural Language Processing (NLP) technique called BERTopic was adopted. This topic modeling pipeline has already been proved efficient and effective in task of topic modelling [57–59].

Given the relatively small text volume, we paid extra attention to pre-processing. The NLTK stopword list was expanded with domain-specific terms (e.g., “juncao”, “technology”, “china”, “cooperation”) and frequent but low-information tokens unique to the data (e.g., “goroka”, “lufa”, “000”, the number stripped off from the whole figures, e.g., 72,000 during tokenization and lemmatization). A normalization procedure was applied to convert all texts to lowercase, generalize high-frequency geographical named entities into the universal token “country” (e.g., “rwanda”, “fiji”, “beijing”), and standardize key domain terms (e.g., harmonizing “jun cao” into “juncao”). These rigorous pre-processing steps served a dual purpose: reducing lexical sparsity, a common issue in small corpora, and minimizing superficial geographic variation, thereby enabling the model to cluster documents based on deeper semantic similarities.

After pre-processing the sequential process of BERTopic was conducted. It involves the following four steps: document embeddings, dimensionality reduction, core clustering and topic labelling. To improve model robustness, several alternative parameter combinations were explored during preliminary analyses, including variations in min_cluster_size, n_neighbors, and embedding configurations. The final parameter settings were selected based on a balance among topic coherence, cluster interpretability, and minimizing the number of documents assigned to outlier clusters. We generated document embeddings using the pre-trained paraphrase-mpnet-base-v2 model, which effectively captures semantic nuances even in relatively small datasets [60]. Dimensionality reduction was then performed with UMAP (n_neighbors = 15, n_components = 5) [61], preserving the essential structure of the embedding space. Core clustering was conducted using HDBSCAN with min_cluster_size = 8, to limit the formation of excessively small clusters and ensure that each topic reflects a substantively meaningful theme. Finally, representative terms for each topic were extracted using the class-based TF-IDF (c-TF-IDF) scheme, a method that extends TF-IDF to quantify term importance at the cluster level, where a higher c-TF-IDF score indicates greater representativeness of the term to its corresponding topic [62]. We computed c-TF-IDF following Grootendorst [63], using the formulation reported in Choi et al. [64]:


$$\begin{array}{c} c-TF-IDF_{i}=\frac{t_{i}}{w_{i}}\times log\frac{m}{\sum_{j}^{n}t_{j}} \end{array}$$

The frequency of wordtis extracted for each classiand divided by the total number of wordsw. Next, the average number of words per classmis divided by the total frequency of wordtacross all classesn.

Topic quality was qualitatively assessed based on three criteria: (1) semantic coherence of representative keywords; (2) interpretability of documents grouped within each cluster; (3) distinctiveness between neighboring topics. Meanwhile, given the relatively small corpus size, all generated clusters were manually reviewed by two researchers, a professor and a lecturer, both specializing in English Language and Literature, to ensure thematic consistency and interpretability. Inter-coder reliability was assessed using Cohen’s kappa statistic, which yielded a value of 0.97, indicating excellent inter-coder reliability.

Final topic labels were assigned based on the top 10 weighted keywords generated for each cluster. The two researchers interpreted topic meanings by examining the most representative keywords and their corresponding weights, with reference to clustered documents where necessary to ensure semantic consistency and interpretability. The two coders independently proposed topic labels and subsequently resolved discrepancies through discussion until consensus was reached.

### 3.3 Emotion analysis

Building upon the thematic patterns identified through BERTopic, emotion analysis was subsequently conducted to examine how these themes were affectively framed in media discourse. While topic modeling reveals what issues are emphasized in Juncao-related news coverage, emotion analysis provides complementary insights into how these issues are presented in terms of emotional orientation. Together, these two analytical components offer a more comprehensive understanding of media framing.

Emotion analysis was conducted to quantify the emotional content embedded in the texts, leveraging NRC Word-Emotion Association Lexicon [48] and NRC Emotion Intensity Lexicon [65], two well-validated lexicons developed by NRC. The NRC Word-Emotion Association Lexicon annotates approximately 14,200 English word types with binary indicators (1 or 0) for ten affective categories, comprising eight basic emotions (Anger, Fear, Anticipation, Trust, Surprise, Sadness, Joy, and Disgust) and two emotion polarities (Positive and Negative), indicating whether each word is associated with a given category. In contrast, the NRC Emotion Intensity Lexicon provides continuous real-valued intensity scores, ranging from 0 to 1, for roughly 6,000 English words.

These two lexicons were jointly applied to the cleaned corpus to obtain both emotion category labels and corresponding intensity scores for emotional expressions. A context-filtering procedure was then implemented to reduce contradictory emotion assignments caused by lexical ambiguity. Sentence-level sentiment polarity was computed using TextBlob. Sentences with negative polarity (polarity < 0) were excluded from positive-emotion categories (Joy, Trust, Anticipation, Surprise), whereas sentences with positive polarity (polarity > 0) were excluded from negative-emotion categories (Anger, Fear, Sadness, Disgust). This step ensured contextual consistency between general sentiment orientation and detected emotion labels. Finally, emotion features were extracted at the sentence level. For each sentence, the frequency of emotion-related words in each category and the corresponding cumulative intensity scores were calculated and used as emotion indicators in subsequent analyses.

### 3.4 Statistical analysis

Sentence-level emotion scores for eight basic emotions (Joy, Trust, Anticipation, Surprise, Anger, Fear, Sadness and Disgust) were analyzed across topics. For each emotion, the assumptions of normality and homogeneity of variances were examined using the Shapiro-Wilk test and Levene’s test, respectively. As the data generally did not meet the assumption of normality, a non-parametric analysis, namely the Kruskal-Wallis test, was used to compare distributions across topics. Benjamini-Hochberg false discovery rate (BH-FDR) correction was used to adjust for multiple comparisons across emotions. For emotions with significant overall differences, Dunn’s post-hoc test with BH-FDR adjustment was performed for pairwise topic comparisons. Effect sizes (ε²) were calculated for the Kruskal-Wallis test to quantify the magnitude of differences across topics.

## 4. Results and analysis

In this section, we present both quantitative and qualitative analyses. We begin with a description of the topics identified by BERTopic, then examine the frequency and intensity of emotion words, and finally explore eight emotion categories, with particular attention to the inter-correlations between topics and emotions through emotional and discourse analyses.

### 4.1 Topic description

**Fig 3:**
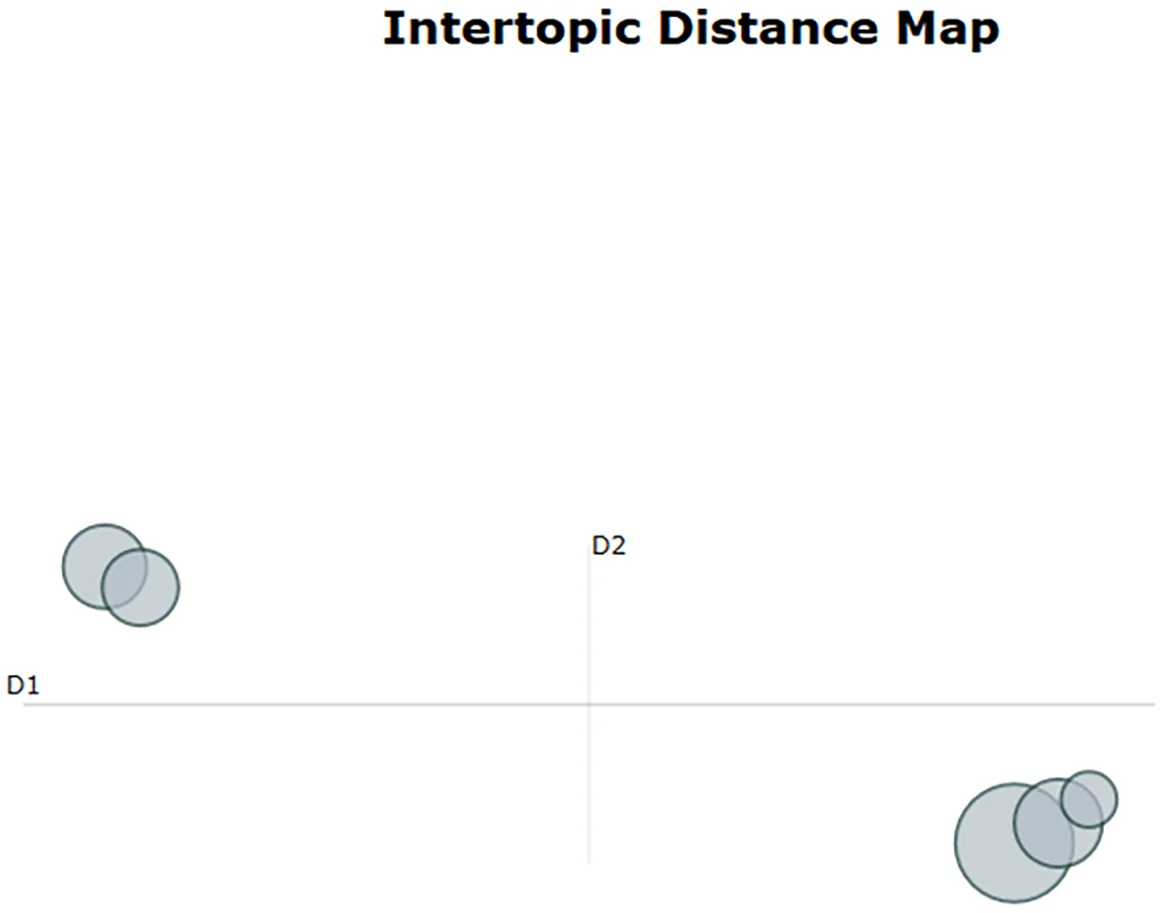
Intertopic distance map generated by BERTopic.

Topic modeling was conducted using BERTopic in Python. The intertopic distance map is presented in Fig 3 and The thematic structure of the corpus is summarized in Table 2.

**Table 2:** Thematic structure of the corpus: Topic labels, representative keywords with weights, and topic proportions. Semantic cluster of keywords, weights and proportion.

Topic	Topic label	Representative keywords with weights	Proportion
**0**	Government-led rice cultivation and farmer training for sustainable production	rice(0.026), farmers(0.015), farming(0.013), training(0.011), district(0.010), production(0.009), government(0.009), province(0.008), sustainable(0.008), produce(0.007)	37.1%
**1**	Local agricultural development, ecological management and poverty alleviation	farmers(0.016), local(0.013), used(0.010), agricultural(0.010), cultivation(0.009), center(0.009), soil(0.009), poverty alleviation(0.008), alleviation(0.008), water(0.008)	20.6%
**2**	The Belt and Road Initiative — International agricultural investment for a shared global community	road(0.015), international(0.012), belt road(0.011), belt(0.011), global(0.010), shared(0.010), initiative(0.009), investment(0.009), agricultural(0.009), community(0.009)	18.6%
**3**	Provincial and intergovernmental officials visit	province(0.025), government(0.020), visit(0.016), provincial(0.013), minister(0.013), governor(0.011), prime(0.011), prime minister(0.011), mp(0.010), park(0.010)	15.5%
**4**	Contributing to UN SDGS through livestock production	sustainable(0.024), increase(0.021), livestock(0.020), un(0.016), goals (0.015), sustainable goals(0.014), farmers(0.013), feed(0.013), milk(0.013), workshop(0.012)	8.2%

**Note.** Proportion represents the percentage of news articles assigned to each topic relative to the entire corpus.

In Fig 3, each bubble represents one topic, the bubble size indicates the proportion of articles assigned to that topic, and the distance between bubbles reflects semantic similarity. The map shows two relatively distinct clusters: Topics 0, 1, and 4 are located in the lower-right quadrant, while Topics 2 and 3 are grouped in the upper-left quadrant. The visual separation between the two groups suggests that the corpus is organized around two main thematic areas.

As shown in Table 2, Topic 0 accounts for 37.1% of the corpus, making it the most prominent topic. Its highest-weighted word is “rice” (0.026), followed by “farmers”, “farming”, “training”, “district”, “production”, “government”, “province”, “sustainable”, and “produce”. These keywords indicate that Topic 0 focuses on government-led rice cultivation and farmer training for sustainable production. Topic 1 represents 20.6% of the corpus and includes words such as “farmers”, “local”, “used”, “agricultural”, “cultivation”, “center”, “soil”, “poverty alleviation”, “alleviation”, and “water”. This topic is associated with local agricultural development, ecological management, and poverty alleviation. In Fig 3, Topics 0 and 1 appear close to each other, indicating a strong thematic connection between agricultural training and local development outcomes.Taken together, Topics 0, 1, and 4 account for 65.9% of the corpus. In Fig 3, these three topics appear in the same cluster, indicating that they are both visually and thematically connected.

Topic 2 accounts for 18.6% of the articles. Its keyword profile includes “road”, “international”, “belt road”, “belt”, “global”, “shared”, “initiative”, “investment”, “agricultural”, and “community”. This pattern identifies Topic 2 as the Belt and Road Initiative and international agricultural investment for a shared global community. Topic 3 represents 15.5% of the corpus and is characterized by terms such as “province”, “government”, “visit”, “provincial”, “minister”, “governor”, “prime”, “prime minister”, “MP” (Member of Parliament), and “park”. These terms indicate that Topic 3 centers on provincial and intergovernmental officials’ visits. In Fig 3, Topics 2 and 3 are positioned close to each other in the upper-left cluster, suggesting that they share a similar thematic orientation related to official exchanges and institutional cooperation.

Topic 4 accounts for 8.2% of the corpus. Its main keywords include “sustainable”, “increase”, “livestock”, “UN”, “goals”, “sustainable goals”, “farmers”, “feed”, “milk”, and “workshop”. This topic reflects contributions to the UN Sustainable Development Goals through livestock production. Although Topic 4 has a smaller proportion than Topics 0 and 1, it is still located within the same broader cluster in Fig 3, indicating its association with practical agricultural application and sustainable production.

Overall, Table 2 shows that Topics 0 and 1 occupy the largest proportions of the corpus, while Fig 3 shows that Topics 0, 1, and 4 are grouped together and separated from Topics 2 and 3. This distribution suggests two main thematic groupings in the corpus: one centered on agricultural application, farmer training, and sustainable production, and the other centered on international cooperation and official exchanges.

### 4.2 Frequency and intensity of emotion words

**Fig 4:**
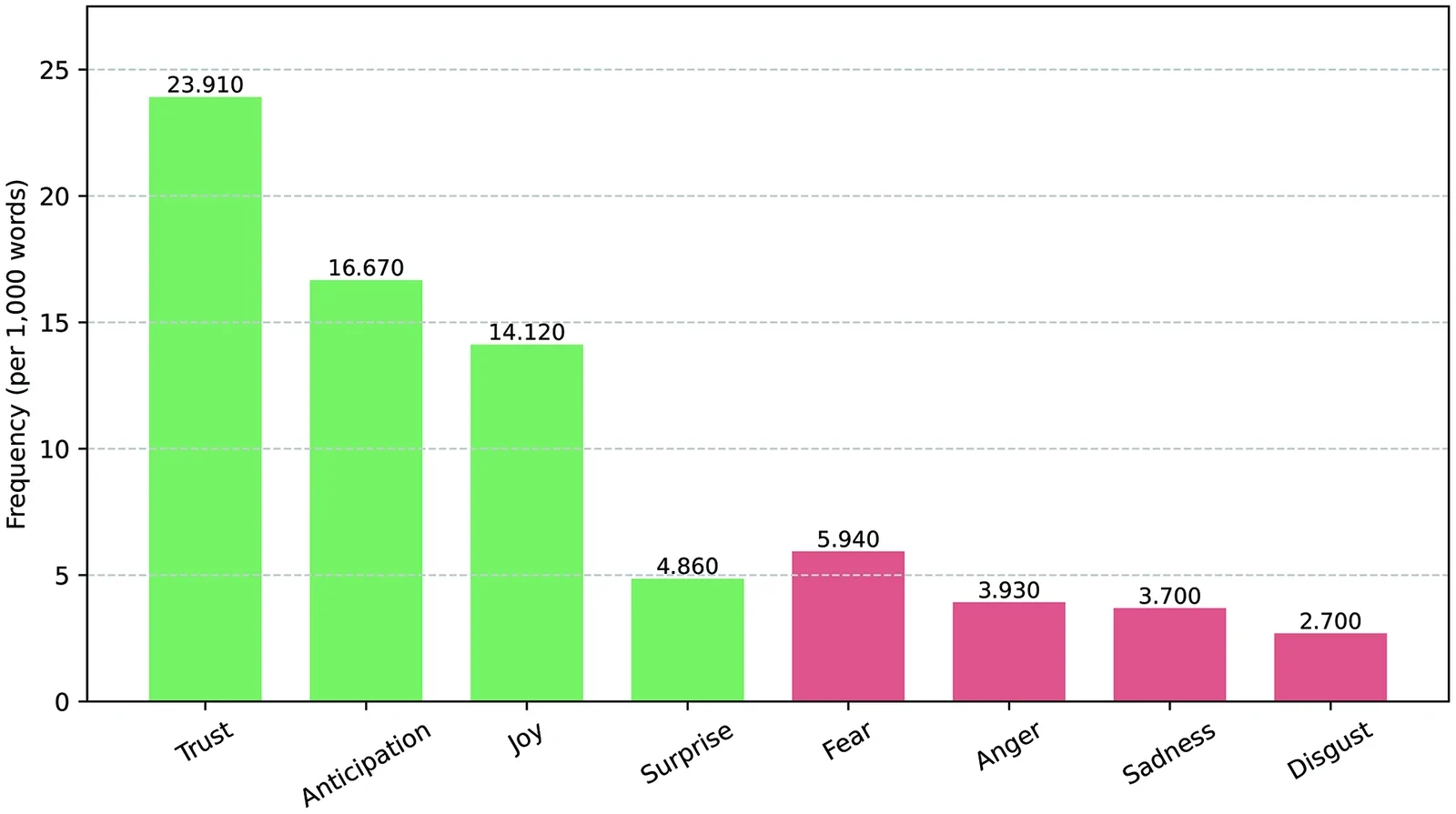
Frequency of emotion words in each emotion category.

Fig 4 shows the normalized frequencies of the eight emotion categories per 1,000 words. Overall, positive emotion categories, shown in green, occur more frequently than negative categories, shown in red. The four positive emotions together account for 59.56 occurrences per 1,000 words, whereas the four negative emotions account for 16.27 occurrences per 1,000 words, indicating that positive emotion terms appear approximately 3.7 times more frequently than negative ones. Among all categories, Trust ranks first with 23.91 occurrences per 1,000 words, followed by Anticipation at 16.67 and Joy at 14.12. Fear is the most frequent negative emotion, with 5.94 occurrences per 1,000 words, ranking above Surprise, which records 4.86 occurrences per 1,000 words. Anger, Sadness, and Disgust occupy the lowest positions, with Disgust being the least frequent overall at 2.70 occurrences per 1,000 words.

**Fig 5:**
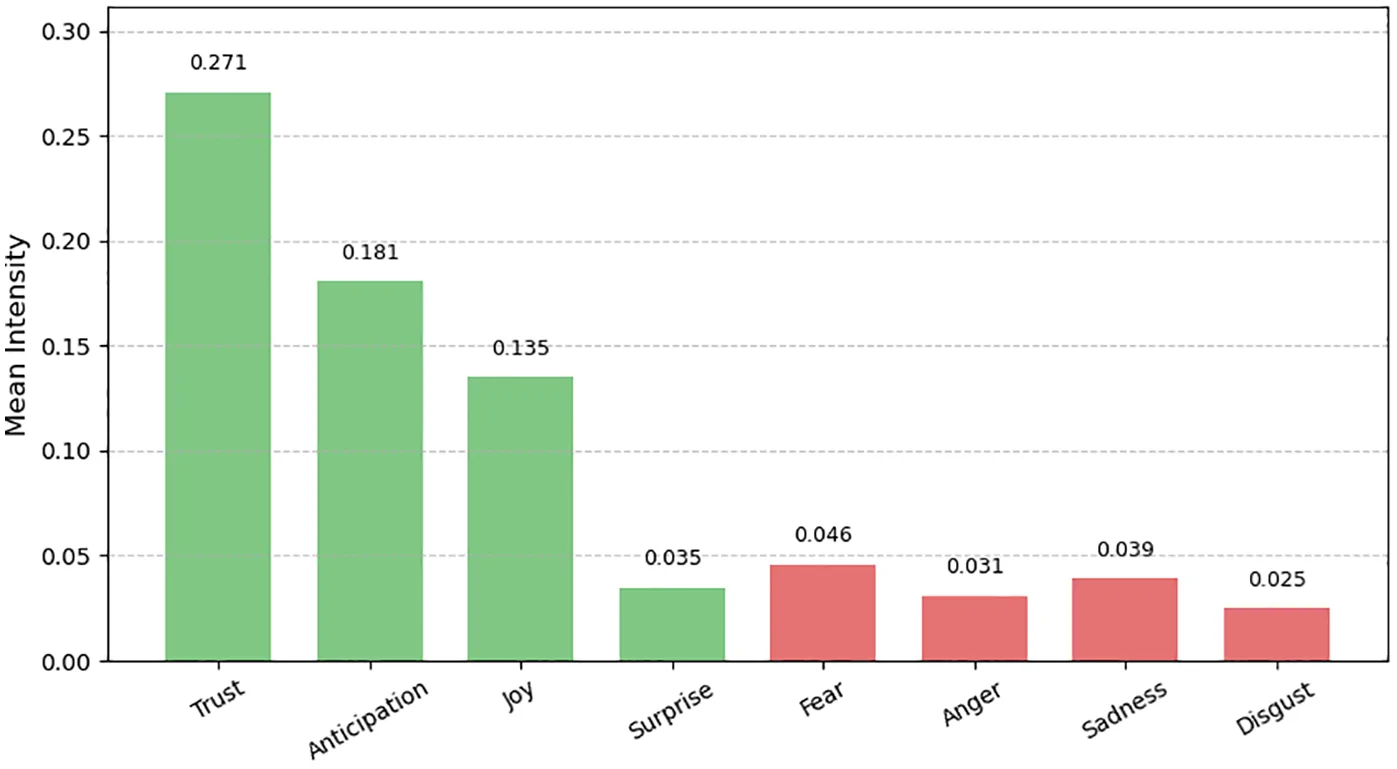
Intensity of each emotion category.

The intensity values for the eight emotions, illustrated in Fig 5, were derived by averaging their respective intensities across all sentences. The results are broadly consistent with the frequency distribution shown in Fig 4. Trust remains the most intense emotion category, with an average intensity of 0.271, followed by Anticipation at 0.181. By contrast, Surprise and the four negative emotions display comparatively lower intensity values, with only minor differences in their respective rankings. This pattern indicates that positive emotions are not only more frequent in the corpus but also more strongly expressed at the sentence level. These statistical results provide the empirical basis for the comparative discussion of Juncao technology and other agricultural technologies in Section 5. In descriptive terms, the Juncao corpus differs from patterns reported in previous studies of gene-edited crops, and digital agriculture [66], as its most frequent emotion categories are concentrated in Trust, Anticipation, and Joy rather than risk- or controversy-related categories.

### 4.3 Emotion categories across topics

The Kruskal-Wallis test identified significant differences among emotion categories across topics (see S1 Appendix). To explore these disparities in depth, Dunn’s post hoc test was conducted, and the results (see S2 Appendix) inform the subsequent analysis structured by individual emotion categories, with special focus on emotion-topic pairs where significant differences were observed. S3 Appendix lists mean emotion intensity of each emotion category by topic. In subsequent analyses, emotion words within each category were ranked according to an intensity index derived by multiplying their frequencies by the corresponding intensity scores provided in the NRC Emotion Intensity Lexicon.

A closer examination of emotion words with high intensity index from Tables 3 and 4 listed below reveals some overlaps in emotion words across categories. For example, *income* occurs in both Anticipation and Joy emotion categories; *good* in Joy and Surprise emotion categories. This phenomenon is even obvious in negative emotions, with *fight* and *combat* appearing in both Fear and Anger, *unfavorable* in both Sadness and Disgust, and *cutting* in all negative categories except Fear.

**Table 3:** Top 5 emotion words by intensity index within the most salient topic in each emotion category.

Emotion	Topic	Word	Frequency	Intensity Scores in NRC	Intensity Index
**Trust**	**2**	cooperation	126	0.711	89.586
		important	15	0.633	9.495
		ambassador	13	0.664	8.632
		trade	18	0.461	8.298
		committed	10	0.805	8.05
**Anticipation**	**0**	production	32	0.469	15.008
		harvest	23	0.578	13.294
		start	13	0.75	9.75
		grow	14	0.555	7.77
		income	15	0.5	7.5
**Joy**	**0-4**	good	36	0.547	19.692
		food	69	0.258	17.802
		income	37	0.403	14.911
		happiness	14	0.984	13.776
		achieve	15	0.641	9.615
**Surprise**	**2**	hope	11	0.367	4.037
		good	13	0.219	2.847
		advance	6	0.375	2.25
		inspired	5	0.406	2.03
		aspiration	5	0.391	1.955

**Table 4:** Top 5 emotion words in topic 4 of each negative emotion category by intensity index.

Emotion	Topic	Word	Frequency	Intensity Scores in NRC	Intensity Index
**Fear**	**4**	poverty	23	0.391	8.993
		insecurity	6	0.484	2.904
		fight	4	0.719	2.876
		government	8	0.359	2.872
		combat	3	0.728	2.184
**Anger**	**4**	poverty	23	0.312	7.176
		demand	11	0.485	5.335
		fight	4	0.667	2.668
		combat	3	0.667	2.001
		cutting	3	0.485	1.455
**Sadness**	**4**	poverty	23	0.69	15.87
		cutting	3	0.422	1.266
		unfavorable	2	0.469	0.938
		shortage	3	0.281	0.843
		starvation	1	0.819	0.819
**Disgust**	**4**	poverty	23	0.469	10.787
		soil	7	0.312	2.184
		cutting	3	0.352	1.056
		crude	2	0.508	1.016
		unfavorable	2	0.414	0.828

Such overlaps are not unexpected, given that the NRC Emotion Intensity Lexicon is employed to assess the intensity of emotion words. The repetition arises from the lexicon’s overlapping mappings, whereby a single lexical item may be associated with multiple emotion categories. Taking *poverty* as a typical example, it is assigned varying intensity scores across negative emotions, ranging from 0.312 in Anger to 0.69 in Sadness. Due to its high frequency, it consistently tops the lists for all negative emotions, as demonstrated in Table 4.

#### 4.3.1 Positive emotion.

Overall, the distribution of positive emotions across the five topics shows some statistically detectable differences, although a few emotions displaying small or negligible effect sizes despite. Trust exhibits the clearest topic-specific variation (H = 52.66, adjusted p < .001, ε² = .022), with Topic 2—the Belt and Road Initiative—standing out from other topics, whereas Anticipation and Joy remain largely uniform across thematic contexts (Anticipation: H = 11.93, adjusted p = .020, ε² = .004; Joy: H = 6.50, adjusted p = .165, ε² = .001). Surprise shows only modest and localized variation, again most evident in Topic 2 (H = 29.64, adjusted p < .001, ε² = .011). Taken together, these results suggest that positive emotional framing is generally stable across topics, with only subtle topic-level modulation rather than pronounced emotional polarization.

Table 3 lists the top 5 emotion words of the highest intensity index within the most salient topic in each emotion category. Given the generally small effect sizes across topics, words were selected from the topics showing relatively stronger differentiation in order to better highlight prototypical emotion expressions. As Joy is the only category exhibiting no significantly difference among five topics, the top five emotion words in this category are selected across all topics.

(1) Trust

The distribution of Trust varied significantly across topics (H = 52.66, adjusted p < .001), although the effect size was small (ε² = .022). Post-hoc comparisons confirmed that Topic 2 differed significantly from the other topics (p < .0001, r = 0.17–0.20). This pattern suggests a modest emphasis on Trust in Topic 2, which may reflect the media’s relatively stronger confidence in developing countries regarding the Belt and Road Initiative.

*Cooperation, ambassador* and *trade* emerge as the primary sources of the emotion Trust, as shown in Table 3. Concordance lines for *cooperation* reveals the frequent recognition of the Belt and Road Initiative as a “cooperation platform” as in Example 1, offering opportunities for “south-south”, “China-African”, and “international” cooperation. These cooperation—primarily in the areas of Juncao technology, agriculture, industrial, and investment—are consistently characterized as high-quality and win-win, with the effect of “poverty reduction”, as in Example 2. Meanwhile, the role of Juncao or Juncao technology is accentuated as *important*—a trust-evoking modifier—given its capability to address concerns locally and globally, as illustrated in Example 3.

Example 1. Since its launch in 2013, the Belt and Road Initiative has been welcomed by the international community as both a public good and a ***cooperation*** platform. [Our Today, Jamaica, 12 October 2023]

Example 2. Juncao technology is a bridge linking Chinese-foreign poverty reduction ***cooperation***, contributing Chinese schemes and wisdom. [Independent Online, South Africa, 30 August 2021]

Example 3. Karuranga said Juncao has played an ***important*** role in building resilience to climate change. “It has also helped combat land degradation by producing fodder for livestock and minimizing soil erosion”. [The Star, Malaysia, 3 August 2024]

(2) Anticipation

Although Anticipation varied significantly across topics (H = 11.93, adjusted p = .020), the effect size was extremely small (ε² = .004), indicating that differences were statistically detectable but practically negligible, and that Anticipation was largely uniform across topics.

High-intensity emotion words listed in Table 3 indicate anticipated enhancements in agricultural output and subsequent economic gains. Terms such as *production, harvest*, and *grow* are predominantly associated with “rice” and “mushrooms”, suggesting increased agricultural productivity following the introduction of dry-rice and Juncao technology, together with the accompanying training programs delivered by Chinese experts, as illustrated in Example 4. Similarly, *start*, frequently co-occurring with “rice farm”, “industry”, and “business”, along with *income*, often collocating with “increase” and “generate”, underscores expectations of enhanced economic resources as illustrated in both Example 4 and 5.

Example 4. According to Karangwa, over 37,000 farmers were trained on Juncao technology and more than 3,800 households and over 50 companies and cooperatives are engaged in mushroom cultivation. He said the technology helped increase mushroom ***production*** compared to the past 15 years. Farmers, he said, can generate ***income*** in seven to 10 days after planting mushrooms, with up to 1,200 kilos of fresh mushrooms produced on 110 square meters of land in a year. [The News Times, Rwanda, 12 August 2024]

Example 5. “... I realized that it requires little capital to ***start*** such a business, on a small size of land. I ***started*** my business with Rw50, 000 as capital,” Umukunzi told Doing Business. [The News Times, Rwanda, 16 August 2024]

(3) Joy

**Fig 6:**
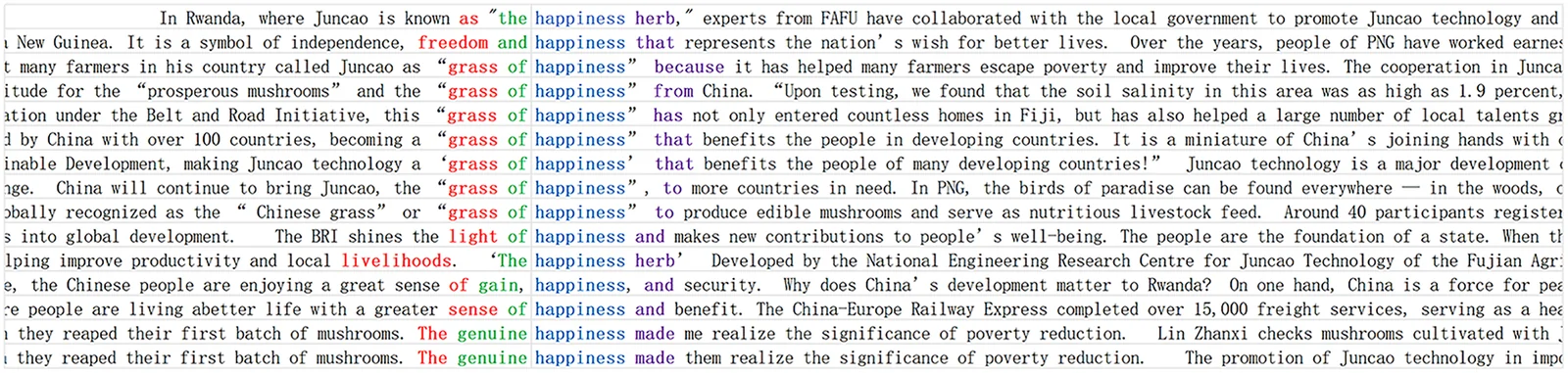
Concordance lines containing *happiness* in the emotion category Joy.

No significant differences were observed in the distribution of Joy across topics (H = 6.50, adjusted p = .165), with a negligible effect size (ε² = .001), indicating a highly consistent expression of Joy across thematic contexts. Among the most frequent Joy-related words, “happiness” (listed in Table 3) is strongly associated with Juncao technology, reflecting the range of benefits it provides. Concordance lines presented in Fig 6 illustrate that Juncao is often described as the “grass of happiness” or “happiness herb”, conveying connotations of prosperity and optimism.

(4) Surprise

Post-hoc comparisons revealed that Surprise in Topic 2 differed only slightly from other topics (r = 0.049–0.110), suggesting statistically detectable but practically modest differences. Despite the small magnitude of these differences, Topic 2 exhibits typical patterns in how Surprise is expressed. To illustrate this, the top five emotion words—hope, good, advance, inspired, and aspiration—are presented. These words in Topic 2 demonstrates that the adoption of Juncao technology within the framework of China’s Belt and Road Initiative brings to local communities both tangible benefits, such as enhancing livelihoods as in Example 6 and intangible benefits, such as instilling hope, fulfilling aspirations, and advancing modernization, as in Example 7–9.

Example 6. Juncao technology allows communities to make a ***good*** living without high up-front costs while having tremendous ecological upside—a panacea for developing economies. [Our Today, Jamaica, 11 November 2021]

Example 7. China’s Juncao technology is powerful  ... It is a “magical tool” of global poverty alleviation that brings ***hope*** of sustainable development to developing countries. [Our Today, Jamaica, 15 September 2021]

Example 8.The Chinese actions to build a community with a shared future for mankind fully demonstrated the country’s sense of responsibility and have turned people’s ***aspiration*** for a better life into reality. [Independent Online, South Africa, 13 October 2022]

Example 9. At his inauguration ceremony, President Kagame said: “Let us work together to advance our cooperation in various fields, and inject new momentum into the comprehensive strategic partnership.” [The News Times, Rwanda, 17 September 2024]

#### 4.3.2 Negative emotions.

As shown in S2 and S3 Appendices, all four negative emotion categories exhibit significantly higher intensity in Topic 4—Contributing to the SDGs of UN through livestock production. Although the effect sizes of negative emotions are generally small across topics (Sadness: ε² = .015; Anger: ε² = .029; Fear: ε² = .009; Disgust: ε² = .042), a notable convergence is observed in Topic 4, where all four negative emotions simultaneously reach their highest relative values. This consistent pattern, despite its modest magnitude, indicates that Topic 4 is associated with a comparatively stronger negative emotional framing than other topics.

Accordingly, the top five emotion words in each category, listed in Table 4, were selected from Topic 4. Notably, *poverty* emerges as the primary source of negative emotions, ranking highest not only in the Fear category but also in Anger, Sadness, and Disgust, as shown in Table 4. This pattern suggests that economic issues constitute the principal concern for developing countries, as indicated in Example 10. The recurrence of *fight* across Fear and Anger further corroborates this concern, as evidenced in Example 11.

Environmental problems represent another factor eliciting these negative emotions. Specifically, the appearance of *cutting* in Anger, Sadness, and Disgust, as well as *unfavorable* in Sadness and Disgust, directly reflects unsatisfactory environmental conditions, as shown in Example 12 and 13.

Example 10. The pace of global ***poverty*** reduction has been slowing down, with the share of the world’s population living in extreme ***poverty*** declining to 8.2% in 2019, from 10% in 2015 and 15.7% in 2010. [Daily News, Botswana, 15 March 2022]

Example 11. The aim was to help African countries address food insecurity and ***fight*** against poverty. [Tanzania Daily News, Tanzania, 27 July 2024]

Example 12. Recognized by the United Nations (UN), this technology addresses food insecurity, generates household income, and creates employment opportunities. It allows small farmers to grow mushrooms using dried, chopped grasses, without resorting to ***tree-cutting*** or environmental harm. [Vanuatu Daily Post, Vanuatu, 4 July 2024]

Example 13. Thus, Juncao technology has the ability to answer and solve those problems by helping farmers to grow food in ***unfavorable*** environments. Juncao technology was founded in the 1980s by Prof Lin Zhanxi of Fujian Agriculture and Forestry University (FAFU). [Tanzania Daily News, Tanzania, 27 July 2024]

(1) Fear

**Fig 7:**
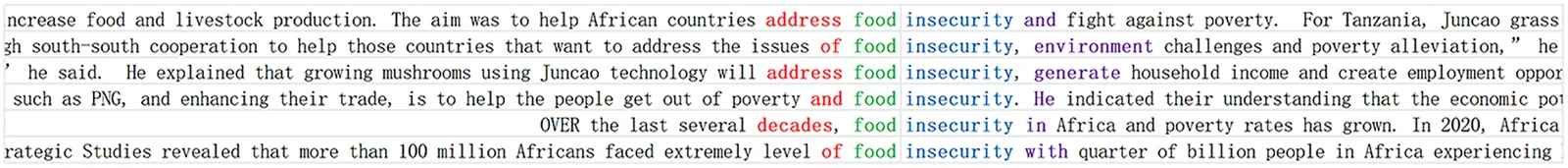
Concordance lines containing *insecurity* in the emotion category Fear.

The emotion term insecurity, which is consistently modified by “food” as illustrated in Fig 7, points to food security as a major threat for developing countries. To address these issues, the SDGs have been established to “fight against poverty” as illustrated in Example 11, and “combat land degradation and desertification”, as illustrated in Example 14.

Example 14.  ... the Juncao grass has been used to ***combat*** land degradation and desertification, conserve water and restore and maintain soil fertility. [Tanzania Daily News, Tanzania, 27 July 2024]

(2) Anger

*Demand* is the emotion word only appears in this emotion category. “Food” and “milk” are frequent collocates of it, which further corroborates the prominence of food security. Additionally, the collocation of “land” with the emotion word *demand* implies that the scarcity of land for agricultural production and the consequent conflicts constitute another trigger for the sense of anger, as substantiated in Example 15. With its capacity to serve as livestock feed, Juncao grass is regarded as a notable example of alleviating land-use conflicts between agriculture and animal husbandry, thereby making a positive contribution to the achievement of the United Nations Sustainable Development Goals (SDGs).

Example 15. It is in fact that, these conflicts are caused by the lack of sufficient land to feed livestock, but also due to the increase of population, and thus, the increase in the ***demand*** for land for farming. [Tanzania Daily News, Tanzania, 27 July 2024]

(3) Sadness

Emotion word *shortage* is frequently associated with “milk” and “forage” as referenced in Example 16, which showcases the challenges faced by the animal husbandry sector.

Example 16. At the same time, The International Farm Comparison Network (IFCN), predicts that, by the year 2030, there will be a ***shortage*** of 6 billion tons of milk worldwide. [Tanzania Daily News, Tanzania, 27 July 2024]

(4) Disgust

Within this emotion category, *soil* points to an environmental problem, as evidenced by its frequent collocation with “erosion”, as illustrated in Example 17. Juncao grass, however, is regarded as the possible solution to this problem.

Example 17. Planting juncao grass in drought prone areas has also been shown to minimise ***soil*** erosion and to combat desertification. [Daily News, Botswana, 15 March 2022]

Overall, negative emotions in Topic 4—Contributing to the SDGs of UN through livestock production—are more prominently associated with references to unsatisfactory global or local conditions, such as poverty, food insecurity, and environmental degradation.

## 5. Discussion

This section aims to explore the thematic and emotional landscape of news media coverage of Juncao technology in developing countries by drawing on media discourse analysis, Hekkert et al.’s Technological Innovation Systems (TIS) framework [67], and Rogers’ innovation diffusion paradigm [29].

### 5.1 The distinctive thematic emphases in news media coverage of Juncao technology in developing countries

One of the main objectives of this study is to explore how news media coverage frames the diffusion of Juncao technology in developing countries. As reported in the results, the thematic distribution shows a clear concentration on application-oriented content, with Topics 0, 1, and 4 together accounting for 65.9% of the corpus. This statistical pattern suggests that media discourse selectively foregrounds Juncao’s practical and developmental value. In this sense, the media does not simply report on the technology; it helps define which aspects of the technology become most visible in public discourse.

The source composition of the corpus helps explain this pattern. Most articles were published by official or state-affiliated agencies, such as the Fiji Government and the Philippine News Agency as well as by major national newspapers that often comply with government agendas, including *The National* and the *Post-Courier*. This suggests that the coverage largely reflects state-led modernization priorities, optimism about technology, and South–South cooperation. Such coverage enables the rapid dissemination of Juncao technology to a large audience [35] and facilitates its adoption. However, this source composition also suggests that the findings should be interpreted with caution, as English-language media coverage may under-represent local-language perspectives and community-level voices. Moreover, regional differences in media accessibility and the visibility of Juncao projects may lead to uneven coverage across developing countries.

The findings related to Topic 0 supports existing adoption theories that stress the importance of extension services and training for smallholder farmers [2,28,29]. Another interesting finding is the frequent mention of staple crops such as “dry rice”, which appears primarily in the context of Papua New Guinea. This suggests that Chinese agricultural technology aid teams not only promote Juncao technology but also introduce locally adapted crop cultivation practices, highlighting the importance of tailoring technology transfer to country-specific agricultural conditions. In Topic 1, the focus on local ecological and socioeconomic improvement, particularly soil and water conservation, aligns with the known benefits of Juncao technology for land recovery, reducing deforestation, and improving livelihoods, which complies with the prior research [5,7,13]. In Topic 2, “the Belt and Road” theme places Juncao technology within the broader context of international investment and South-South cooperation [15]. Furthermore, in Topic 3, the frequent reporting on governmental engagements such as ministerial and provincial visits suggests that these official interactions play an important role in helping mobilize resources, and connecting policies across different government levels, an aspect that has not been fully explored in previous studies. Notably, Topic 4 shows that the SDGs-aligned livestock theme expands the traditional focus of Juncao technology beyond mushroom cultivation [12]. The repeated mentions of “feed” and “milk” further demonstrate Juncao technology’s role in integrating crop and livestock farming, improving nutrition, and promoting sustainable production, thereby contributing to SDGs 2, 12, and 15.

Overall, the convergence of these five topics suggests that news media coverage does not merely act as a passive information carrier but serves as a constitutive component of TIS in the diffusion of Juncao technology. Drawing on the framework of Hekkert et al. [67], which identifies seven key systemic functions (F1–F7), these findings reveal a complex interplay of systemic drivers within the media discourse. Specifically, the media’s emphasis on technical training (Topic 0) instantiates Knowledge Diffusion through Networks (F3), while its alignment with local ecological management and global SDG mandates (Topics 1 and 4) provides the Guidance of the Search (F4), offering both the “how-to” and the “why” for local adoption. Furthermore, the focus on international investment under the Belt and Road Initiative (Topic 2) serves to operationalize Resource Mobilization (F6), highlighting the financial and material support essential for technology transfer. Perhaps most significantly, the political visibility identified in Topic 3, featuring high-level diplomatic visits, performs the vital function of Creation of Legitimacy (F7). This political endorsement is crucial, as it builds institutional trust and buffers the technology against the inherent risks and resistances associated with new agricultural transitions. Collectively, this media-driven narrative constructs a robust discursive infrastructure, elevating Juncao technology from a single agricultural product to a comprehensive developmental solution that is integrated into the recipient countries’ broader socio-economic and ecological agendas.

### 5.2 Emotional features in news media coverage of Juncao technology in developing countries

Emotions represent an essential component of agricultural technology adoption [11]. Integrating emotional factors into Rogers’ [29] Diffusion of Innovations framework can enrich the model from a psychological perspective. Although statistical tests in the current study indicate only small effect sizes in the distribution of emotions across topics, qualitative discourse analysis reveals typical patterns in how emotions are mobilized within specific thematic contexts.

#### 5.2.1 Constructing positive emotional narratives surrounding technology diffusion.

As evidenced by the high frequency and intensity of positive emotions identified in Section 4.2 (see Figs 4 and 5), a key finding of this study is the strategical mobilization of Trust and Anticipation to catalyze technology diffusion. The peak intensity of Trust (0.271), as illustrated in Fig 5, provides an emotional foundation for international cooperation, particularly the Belt and Road Initiative (Topic 2). Words such as *cooperation, ambassador*, and *trade* in Table 3 frame Juncao technology not merely as an agricultural tool, but also as a symbol of a reliable and “win-win” partnership. This framing is powerfully reinforced through persuasive metaphors, such as describing Juncao as a “bridge”, as illustrated in Example 2, which builds trust by symbolizing connection and mutual support between the Chinese government and other countries. The case of Rwanda underscores how media narratives bridge the gap between technical solutions and strategic goals. For instance, the discourse shifts from an economist’s focus on mitigating land degradation (Example 3) to the President’s vision of broader international cooperation (Example 9). Such framing highlights the institutional dimensions of technology adoption [2], indicating that media narratives help build public anticipation and trust in the political frameworks governing the technology [68]. In this context, institutional trust acts as a critical foundation; without confidence in the delivering actors and their international networks, the adoption of the innovation itself would likely face greater resistance.

In addition, Anticipation is prominently mobilized in narratives concerning rice cultivation and farmer training (Topic 0), aligning with one of Rogers’ [29] core perceived attributes of innovation adoption: Relative Advantage. The media narrative, rich with terms such as *production, harvest, income,* and *start*, actively constructs the expectation of tangible economic benefits, thereby reflecting the economic constraints paradigm. This also indicates that agricultural innovations should meet the local needs of farmers. By showcasing success stories of increased income and mushroom production, as seen in Examples 4 and 5, media discourse provides a clear vision of the Juncao technology’s profitability and its advantage over existing practices, both of which are crucial to its adoption [22]. This focus on economic outcomes serves as a driver of adoption catering to the needs of local farmers in developing countries [2].

Meanwhile, other positive emotions help break down barriers to adoption and fill a gap identified in the literature [34]. For instance, the consistent framing of Juncao as a source of Joy—the “grass of happiness”—enhances its Compatibility [29] with the universal desire for well-being. This narrative aligns the technology with the fundamental values and needs of local communities. The emotion of Surprise, conveyed through terms such as “*magical tool*” and *hope*, serves to reduce the technology’s perceived Complexity [29]. By presenting Juncao technology as a powerful and accessible solution, the media makes it seem less intimidating and enhances its Observability [29] by creating memorable success stories that can be easily disseminated [32].

The predominance of positive emotional framing identified in the present study also provides an important comparative perspective on agricultural technology communication. Existing studies on media coverage of genetically modified or gene-edited crops suggest that agricultural technologies are not necessarily framed according to the “bad news hypothesis”, which assumes that controversy and risk dominate news reporting [66]; instead their news discourse was overwhelmingly pro-innovation centered, framing the technology primarily as an efficient scientific solution to agricultural challenges. However, such positive framing was also criticized for relying heavily on elite sources, including scientists and government officials, while consumer perspectives and opposing voices remained relatively underrepresented [69,70].

In comparison, although Juncao-related coverage in the present study is likewise predominantly positive, its framing is constructed through a broader range of social actors. In addition to scientific and governmental discourse, local farmers and first-hand adopters also participate in the construction of media narratives, as illustrated in Example 5. Such bottom-up narratives provide experiential legitimacy by emphasizing practical benefits, including low investment costs and income-generation opportunities. Moreover, unlike media framing of genetically modified or gene-edited crops, which often centers on technological efficiency, crop productivity, and food security [70], Juncao-related positive framing is embedded within a broader developmental discourse. The technology is associated not only with agricultural productivity, but also with poverty alleviation, ecological sustainability, land restoration, international cooperation, and the Sustainable Development Goals (SDGs). This broader narrative orientation positions Juncao not merely as an agricultural innovation, but as a socially embedded development initiative.

These findings suggest that agricultural technology diffusion is shaped not only by stakeholder coordination, but also by how technologies are emotionally and socially represented in media discourse. In the case of Juncao, the integration of top-down institutional narratives with bottom-up farmer experiences contributes to a more socially grounded and development-oriented communication model, which may facilitate favorable perceptions surrounding technology diffusion.

#### 5.2.2 Using emotion to frame problems and position Juncao technology as the solution.

The analysis of negative emotion in this study reveals that poverty-related concerns in developing countries are primarily reflected in three key livelihood challenges: food insecurity, land shortage, and land degradation. These issues emerge as dominant sources of negative sentiment in media discourse, indicating persistent structural constraints on rural development and agricultural sustainability.

A significant finding concerns how negative emotions are deployed through media discourse. Instead of being associated with Juncao technology itself, negative emotions such as Fear, Anger, Sadness, and Disgust are primarily associated with the critical challenges addressed by the UN SDGs (Topic 4). These emotions are primarily triggered by *insecurity* (Fear; see Fig 7), “land demand” conflicts (Anger; Example 15), *shortage* in animal husbandry (Sadness; Example 16), and environmental degradation such as “soil erosion” (Disgust; Example 17).

This narrative strategy is particularly salient from a media diffusion perspective: the media first vividly portrays these negative conditions and then immediately positions Juncao technology as a potential solution, which may arouse empathetic feelings and generate supportive responses [71]. For instance, Juncao technology is shown to “fight against poverty”, prevent *cutting* of trees, and enable farming in “unfavorable environments” (Example 11–13). This problem-solution framing contributes to enhancing Juncao’s perceived Relative Advantage and its Compatibility with the urgent needs and development goals of the audience’s social system [29,72], thereby creating an emotional urgency for change and solidifying Juncao’s position as an ideal solution. This mechanism establishes a reversed logical path compared to conventional agricultural innovation communication. As Mohr and Höhler [35] observed in digital agriculture, negative media arguments—such as infrastructure deficiency and data security—are typically treated as intrinsic barriers that impede the technology itself, thereby generating adoption anxieties. In sharp contrast, negative narratives in Juncao corpus do not function as implementation barriers; instead, external societal crises (poverty and ecological degradation) are discursively harnessed as the rhetorical justification and catalyst for the innovation, reinforcing its absolute necessity. This finding contributes to the current literature by demonstrating how the strategic framing of negative emotions in media discourse can construct the necessity and value of an innovation, thereby addressing a gap in prior research that has largely overlooked this persuasive mechanism [32,35].

Consequently, in communicating agricultural technologies, it is important to frame technological initiatives in relation to the concrete livelihood challenges faced by developing countries. Effective communication should first acknowledge pressing issues such as food insecurity, land shortage, and land degradation, and then clearly articulate how specific agricultural technologies can address these constraints. Presenting technology solutions in problem-oriented and context-sensitive language can enhance perceived relevance, strengthen local acceptance, and ultimately facilitate more successful technology diffusion.

### 5.3 The synergy of thematic salience and affective framing in technology selection

The analytical convergence of thematic distribution and emotional intensity demonstrates that the media’s role in diffusing Juncao technology is significant in shaping technology selection. This mechanism operates through the alignment between thematic salience, which provides the cognitive basis for selection, and affective framing, which provides psychological reassurance. The statistical concentration of media attention on application-oriented themes, together with the dominance of positive emotional framing, suggests that Juncao technology is repeatedly presented as practical, trustworthy, and developmentally relevant.

At the cognitive level, the thematic salience of practical empowerment and international capacity building (Topics 0 and 1) performs the “Guidance of the Search” function within the TIS framework [67], narrowing down the choice set for external stakeholders. Crucially, this cognitive guidance is dynamically paired with a high-intensity affective priming of Trust and Anticipation. While thematic frames provide the rational justification for selection (e.g., eco-governance and SDGs), the dominant positive emotions provide the affective security needed to overcome the inherent uncertainties of cross-border agricultural transitions.

The structural significance of this interaction lies in what we conceptualize as the “emotional buffering” effect in technology selection. Unlike conventional agricultural innovations that often trigger technological-exclusion or corporate-monopoly anxieties in media discourses [35], the media framing of Juncao technology utilizes positive affective resonance as an emotional shock-absorber. By leveraging pre-existing societal crises as catalysts for adoption (as demonstrated in Section 5.2.2) and buffering technical risks through an infrastructure of Trust, this dynamic interplay elevates Juncao technology from a mere biological innovation to a legitimized developmental solution, accelerating its global selection.

## 6. Conclusion

This study provides new insights into the communicative mechanisms underpinning the adoption of Juncao technology in developing countries through an emotion analysis of news media coverage. The core finding is that media discourse predominantly employs positive emotions, while centering on five key thematic areas. This emotional and thematic framing in news reporting serves not merely as a vehicle for emotion, but also as an indicator of a technology’s diffusion potential, thereby extending the value of linguistic research to technology dissemination practices.

### 6.1 Summary of findings

The study revealed a strategic deployment of emotions. Positive emotions, including Trust, Anticipation, Joy, and Surprise, dominate the narrative, and actively shape the perceived perceptions of Juncao technology. Trust is built through narratives of international cooperation, while Anticipation underscores tangible economic benefits. Joy and Surprise reduce perceived complexity and enhance observability, making the technology desirable. Conversely, negative emotions, including Fear, Anger, Sadness, and Disgust, are strategically adopted to describe pre-existing problems such as poverty and food insecurity, which Juncao technology is positioned to solve. This problem-solution framing amplifies the technology’s relative advantage and compatibility, fostering an emotional interest in learning about Juncao technology, which may facilitate its adoption. This finding implies that effective agricultural communication should connect technological attributes with the lived problems of target communities, thereby transforming abstract technical advantages into locally meaningful and emotionally resonant messages.

### 6.2 Theoretical and practical contributions

Theoretically, this study contributes to the understanding of technology diffusion by integrating an emotional dimension into Rogers’ [29] Diffusion of Innovations framework. While traditional Rogersian models prioritize cognitive evaluations of innovation attributes, our analysis reveals that linguistic strategies and emotional cues in news media are fundamental to constructing these perceptions and catalyzing early-stage adoption. It suggests that the management of innovation is not merely a technical endeavor but a psychologically grounded process that can support broader sustainability transitions. Furthermore, the study demonstrates that the five identified media themes closely echo the functional dimensions of the TIS framework [67]. Specifically, the media’s focus on technical training, global SDG mandates, and high-level diplomatic visibility serves to operationalize knowledge diffusion through networks (F3), guidance of search (F4), and resource mobilization (F6), and the creation of legitimacy (F7). In doing so, it illustrates how media narratives build a favorable discursive foundation, allowing agricultural technology to move from an isolated technical tool to a multidimensional developmental solution aligned with recipient countries’ broader socio-economic and ecological priorities.

Practically, these findings suggest that the international promotion of Juncao technology and similar agricultural innovations depends on a strategic shift toward stakeholder-inclusive and problem-oriented communication. Beyond the specific case of Juncao technology, this study highlights broader implications for agricultural communication and technology dissemination: successful communication strategies should combine technical explanation, emotional framing, attention to local problems, and stakeholder credibility to enhance public acceptance and support the diffusion of agricultural innovations. Future initiatives should leverage media narratives to highlight the integration of governments, research institutions, and farmers, thereby reflecting a balanced synergy of top-down legitimacy and bottom-up agency. To enhance credibility and local acceptance, communication strategies must be explicitly grounded in the concrete livelihood challenges of recipient countries, such as food insecurity and land degradation, positioning technological solutions as direct responses to these context-specific, emotionally resonant challenges.

Furthermore, practitioners should refine their linguistic strategies for different adopter categories [29]. For instance, to engage “early majority” [29] adopters, who seek clear benefits, the communication should continue to emphasize benefit-oriented narratives, such as income generation and improved production. For “laggards” [29] or those still hesitant, communication strategies should incorporate “risk mitigation language”, directly addressing fears and uncertainties by demonstrating how agricultural technology can address existing problems. This will ultimately facilitate more sustainable technology diffusion across developing countries. More generally, the findings indicate that successful agricultural technology dissemination requires communication systems that are emotionally engaging, institutionally credible, locally adaptive, and responsive to the concerns of different adopter groups.

### 6.3 Limitations and future research

A primary limitation of this study is the limited size of the textual corpus. Relying solely on news media sources limits the analysis to publicly disseminate information. Future research could broaden the corpus to include social media, policy documents, or direct interviews to collect more diverse perspectives and emotional expressions. Despite these limitations, this study lays a crucial foundation for understanding the intricate relationship between emotional language in media and the successful diffusion of sustainable agricultural innovations.

AI Use Disclosure.The authors used generative AI tools, namely ChatGPT and DeepSeek, to assist with English language editing and grammatical improvement. All AI-assisted text was reviewed and approved by the authors. All intellectual content, analysis, and interpretations are the authors’ own, and the authors take full responsibility for the manuscript.

## Supporting information

S1 Appendix Results of Kruskal-Wallis tests and BH-FDR adjusted p-values. (DOCX)

S2 Appendix BH-adjusted p-values for Dunn’s post hoc pairwise comparisons of emotion intensities across topics. (DOCX)

S3 Appendix Mean emotion intensity of each emotion category by topic. (DOCX)
