# Supplementary material for: Media framing of agricultural technology adoption in developing countries: Evidence from Juncao technology news coverage
Source: PLoS One. 2026 Sep 1;21(9):e0343652. doi: 10.1371/journal.pone.0343652 (PMC13533395; doi:10.1371/journal.pone.0343652)
Supplement: Supplementary file 1 [file pone.0343652.s001.docx]

**S1 Appendix. Results of Kruskal-Wallis tests and BH-FDR adjusted p-values**

| **Emotion** | **Statistics** | **Adjusted P-values** | **Significance** | **Effect_Size** |
| --- | --- | --- | --- | --- |
| Joy | 6.496233983 | 0.165027828 | FALSE | 0.001105996 |
| Sadness | 37.6471595 | 2.64994E-07 | TRUE | 0.014907913 |
| Anger | 68.3406722 | 2.03352E-13 | TRUE | 0.028507165 |
| Anticipation | 11.92908178 | 0.020441711 | TRUE | 0.003513107 |
| Trust | 52.6572634 | 2.68045E-10 | TRUE | 0.02155838 |
| Surprise | 29.63801957 | 9.27866E-06 | TRUE | 0.011359335 |
| Fear | 23.85840067 | 0.000113691 | TRUE | 0.008798582 |
| Disgust | 98.11633562 | 1.98095E-19 | TRUE | 0.04169975 |
