# Supplementary material for: Media framing of agricultural technology adoption in developing countries: Evidence from Juncao technology news coverage
Source: PLoS One. 2026 Sep 1;21(9):e0343652. doi: 10.1371/journal.pone.0343652 (PMC13533395; doi:10.1371/journal.pone.0343652)
Supplement: Supplementary file 2 [file pone.0343652.s002.docx]

**S2 Appendix. BH-adjusted p-values for Dunn’s post hoc pairwise comparisons of emotion intensities across topics**

| **Trust** | | | | | |
| --- | --- | --- | --- | --- | --- |
| **Topic** | **0** | **1** | **2** | **3** | **4** |
| **0** | 1 | 0.693589555 | 4.72137E-09*** | 0.896216698 | 0.693589555 |
| **1** | 0.693589555 | 1 | 9.87862E-08*** | 0.693589555 | 0.896216698 |
| **2** | 4.72137E-09*** | 9.87862E-08*** | 1 | 8.60861E-06*** | 4.63473E-05*** |
| **3** | 0.896216698 | 0.693589555 | 8.60861E-06*** | 1 | 0.693589555 |
| **4** | 0.693589555 | 0.896216698 | 4.63473E-05*** | 0.693589555 | 1 |
| **Anticipation** | | | | | |
| **Topic** | **0** | **1** | **2** | **3** | **4** |
| **0** | 1 | 0.059169198 | 0.249038993 | 0.034481807* | 0.839439062 |
| **1** | 0.059169198 | 1 | 0.39374113 | 0.337582294 | 0.192116956 |
| **2** | 0.249038993 | 0.39374113 | 1 | 0.14980969 | 0.39374113 |
| **3** | 0.034481807* | 0.337582294 | 0.14980969 | 1 | 0.059169198 |
| **4** | 0.839439062 | 0.192116956 | 0.39374113 | 0.059169198 | 1 |
| **Joy** | | | | | |
| **Topic** | **0** | **1** | **2** | **3** | **4** |
| **0** | 1 | 0.570517909 | 0.533097901 | 0.327603166 | 0.533097901 |
| **1** | 0.570517909 | 1 | 0.327603166 | 0.476610448 | 0.382530345 |
| **2** | 0.533097901 | 0.327603166 | 1 | 0.238178826 | 0.899965046 |
| **3** | 0.327603166 | 0.476610448 | 0.238178826 | 1 | 0.238178826 |
| **4** | 0.533097901 | 0.382530345 | 0.899965046 | 0.238178826 | 1 |
| **Surprise** | | | | | |
| **Topic** | **0** | **1** | **2** | **3** | **4** |
| **0** | 1 | 0.943846176 | 0.0004788*** | 0.433341924 | 0.116746352 |
| **1** | 0.943846176 | 1 | 0.0004788*** | 0.433341924 | 0.116746352 |
| **2** | 0.0004788*** | 0.0004788*** | 1 | 0.111372029 | 1.67891E-05*** |
| **3** | 0.433341924 | 0.433341924 | 0.111372029 | 1 | 0.0708737 |
| **4** | 0.116746352 | 0.116746352 | 1.67891E-05*** | 0.0708737 | 1 |
| **Fear** | | | | | |
| **Topic** | **0** | **1** | **2** | **3** | **4** |
| **0** | 1 | 0.101322695 | 0.108845692 | 0.125884318 | 1.1154E-05*** |
| **1** | 0.101322695 | 1 | 0.900699233 | 0.870152709 | 0.002949447** |
| **2** | 0.108845692 | 0.900699233 | 1 | 0.870152709 | 0.002935268** |
| **3** | 0.125884318 | 0.870152709 | 0.870152709 | 1 | 0.036329822* |
| **4** | 1.1154E-05*** | 0.002949447** | 0.002935268** | 0.036329822* | 1 |
| **Anger** | | | | | |
| **Topic** | **0** | **1** | **2** | **3** | **4** |
| **0** | 1 | 0.000495061*** | 0.020336416* | 0.682661817 | 2.34388E-14*** |
| **1** | 0.000495061*** | 1 | 0.264099862 | 0.03332753* | 1.24945E-06*** |
| **2** | 0.020336416* | 0.264099862 | 1 | 0.196578333 | 7.59156E-09*** |
| **3** | 0.682661817 | 0.03332753* | 0.196578333 | 1 | 7.59156E-09*** |
| **4** | 2.34388E-14*** | 1.24945E-06*** | 7.59156E-09*** | 7.59156E-09*** | 1 |
| **Sadness** | | | | | |
| **Topic** | **0** | **1** | **2** | **3** | **4** |
| **0** | 1 | 0.001323506** | 0.507255597 | 0.360789616 | 5.16321E-07*** |
| **1** | 0.001323506** | 1 | 0.011032932* | 0.191025847 | 0.012208349* |
| **2** | 0.507255597 | 0.011032932* | 1 | 0.602117177 | 5.4433E-06*** |
| **3** | 0.360789616 | 0.191025847 | 0.602117177 | 1 | 0.001323506** |
| **4** | 5.16321E-07*** | 0.012208349* | 5.4433E-06*** | 0.001323506** | 1 |
| **Disgust** | | | | | |
| **Topic** | **0** | **1** | **2** | **3** | **4** |
| **0** | 1 | 1.15025E-09*** | 0.171810831 | 0.593717086 | 8.0142E-16*** |
| **1** | 1.15025E-09*** | 1 | 3.59255E-06*** | 9.76946E-05*** | 0.000900172*** |
| **2** | 0.171810831 | 3.59255E-06*** | 1 | 0.593717086 | 3.31891E-12*** |
| **3** | 0.593717086 | 9.76946E-05*** | 0.593717086 | 1 | 1.15025E-09*** |
| **4** | 8.0142E-16*** | 0.000900172*** | 3.31891E-12*** | 1.15025E-09*** | 1 |

**Note.** The table presents a p-value matrix for pairwise comparisons of emotion intensities across topics using Dunn’s post hoc test. P-values are adjusted using the Benjamini-Hochberg false discovery rate (BH FDR) method. *, **, and *** indicate p ≤ 0.05, p ≤ 0.01, and p ≤ 0.001, respectively.
