# Supplementary material for: Media framing of agricultural technology adoption in developing countries: Evidence from Juncao technology news coverage
Source: PLoS One. 2026 Sep 1;21(9):e0343652. doi: 10.1371/journal.pone.0343652 (PMC13533395; doi:10.1371/journal.pone.0343652)
Supplement: Supplementary file 3 [file pone.0343652.s003.docx]

**S3 Appendix. Mean emotion intensity of each emotion category by topic**

| **Emotion** | **Topic** | **Mean Intensity** |
| --- | --- | --- |
| **Joy** | 0 | 0.126390572 |
|  | 1 | 0.129783276 |
|  | 2 | 0.150751244 |
|  | 3 | 0.115967442 |
|  | 4 | 0.143628788 |
| **Sadness** | 0 | 0.013685185 |
|  | 1 | 0.056668942 |
|  | 2 | 0.030412935 |
|  | 3 | 0.026623256 |
|  | 4 | 0.08405303 |
| **Anger** | 0 | 0.009861953 |
|  | 1 | 0.035493174 |
|  | 2 | 0.031391376 |
|  | 3 | 0.016688372 |
|  | 4 | 0.07932197 |
| **Anticipation** | 0 | 0.201494949 |
|  | 1 | 0.165513652 |
|  | 2 | 0.18115257 |
|  | 3 | 0.14390698 |
|  | 4 | 0.199386364 |
| **Trust** | 0 | 0.245309764 |
|  | 1 | 0.249653584 |
|  | 2 | 0.334127695 |
|  | 3 | 0.239990698 |
|  | 4 | 0.254674242 |
| **Surprise** | 0 | 0.027430976 |
|  | 1 | 0.032634812 |
|  | 2 | 0.054092869 |
|  | 3 | 0.037851163 |
|  | 4 | 0.015420455 |
| **Fear** | 0 | 0.027545455 |
|  | 1 | 0.047307167 |
|  | 2 | 0.047913765 |
|  | 3 | 0.047311628 |
|  | 4 | 0.078356061 |
| **Disgust** | 0 | 0.005195286 |
|  | 1 | 0.040080205 |
|  | 2 | 0.016767828 |
|  | 3 | 0.01232093 |
|  | 4 | 0.062007576 |
